# Integrative genomic analyses support the division of the extended *Asfarviridae* clade into multiple viral families

**DOI:** 10.1128/jvi.01337-25

**Published:** 2025-11-13

**Authors:** Thiago Mendonça-Santos, Jônatas Abrahão, Luiz-Eduardo Del-Bem

**Affiliations:** 1 Graduate Program in Bioinformatics, Institute of Biological Sciences (ICB), Federal University of Minas Gerais (UFMG) 28114, Belo Horizonte, Minas Gerais, Brazil; 2 Laboratório de Medicina e Saúde Pública de Precisão (MeSP2 ), Instituto Gonçalo Moniz (IGM), Fundação Oswaldo Cruz (FIOCRUZ) 37903, Salvador, Bahia, Brazil; 3 Department of Microbiology, Federal University of Minas Gerais (UFMG) 28114, Belo Horizonte, Minas Gerais, Brazil; 4 Department of Genetics, Luiz de Queiroz College of Agriculture (ESALQ), University of São Paulo (USP) 54538 , Piracicaba, São Paulo, Brazil; Indiana University Bloomington, Bloomington, Indiana, USA

**Keywords:** phylogenomics, viral taxonomy, pangenomics, giant viruses

## Abstract

Giant viruses continue to challenge our understanding of virology, blurring boundaries of what a virus can be. The so-called “extended *Asfarviridae*”— such as African swine fever virus, faustovirus, kaumoebavirus, pacmanvirus, and AbALV—has long puzzled taxonomists. By integrating comparative genomics, pangenomics, and phylogenomics, we show these lineages are deeply divergent, forming multiple families rather than one. This work underscores the huge unexplored diversity of giant viruses and demonstrates the value of integrative genomic analyses for proper taxonomic delineation.

## INTRODUCTION

Giant viruses, such as mimiviruses*,* exhibit unique characteristics, including virions visible under a light microscope, genomes larger than some unicellular organisms, and a diverse and unprecedented gene repertoire among viruses (1, 2). Their discovery revolutionized virology, challenging established concepts, underscoring the need to revisit conventional definitions, and highlighting the necessity for new isolation and sequencing studies to discover more of these long-overlooked viruses.

The African swine fever virus (ASFV), one of the largest known DNA viruses before the discovery of other giant viruses, remained the sole representative of the *Asfarviridae* family for decades and is the causative agent of African swine fever (ASF). However, other genetically related viruses have been isolated and placed within the *Asfarviridae* clade, leading to the formation of the “extended *Asfarviridae”* group to include ASFV and genetically related viruses such as Faustovirus, Kaumoebavirus, Pacmanvirus, and Abalone asfar-like virus (AbALV). Faustovirus, isolated in 2015 from wastewater (3), and kaumoebavirus, discovered in 2016 (4), both infect *Vermamoeba vermiformis*. Pacmanvirus, identified in 2017 from wastewater (5), replicates in *Acanthamoeba castellanii*. AbALV was first identified in 2020 and is known to infect abalones, causing high mortality rates (6).

Despite the apparent genetic relationship, there is still uncertainty about the inclusion of these associated viruses within the family *Asfarviridae* due to highly divergent characteristics such as differences in host range, environmental niches, genome sizes, and gene repertoire. Questions persist about whether these viruses should remain grouped under a single family or if they represent lineages distinct enough to warrant classification as founding members of entirely new viral families.

To address these issues, we performed comprehensive genomic and functional analyses and constructed a pangenome that reflects the gene content of the extended *Asfarviridae*. This approach provides an *in silico* framework for investigating the evolutionary relationships among these viruses, exploring their genetic similarities and differences, and characterizing their complex genomic architecture and functional diversity. By addressing this knowledge gap, our work contributes to a more precise taxonomic classification while revealing aspects of the genetic mechanisms that underlie their diversity, evolution, and environmental adaptation.

## METHODOLOGY

### Genome acquisition and gene prediction

Publicly available genomes of viral isolates from members of the extended *Asfarviridae* clade were downloaded from the NCBI database. Only complete genomes were downloaded. Gene prediction was performed for each of these genomes to eliminate gene prediction method bias and ensure methodological homogeneity. The genomes were submitted to GeneMarkS v4.28 (7) for the prediction of coding regions.

### Predicted protein sequence clustering

To determine the families of orthologous genes among different viral species, the amino acid sequences of predicted proteins were grouped into clusters of potentially orthologous elements using the Proteinortho v6.0.14 tool (8). The parameters of 60% coverage and 20% identity for protein clustering were adopted based on a previous study (5). The pangenome was categorized according to the level of gene sharing as follows: Core, which includes genes present in 100% of the genomes; Soft-core, which includes genes present in 90% of the genomes; Shell, which includes genes present in three or more genomes but less than 90% of the genomes; and Cloud, which includes genes present in two or one genomes. The simplified representation of the pangenome was visualized through a flower plot generated using the custom script available at https://github.com/thiago22yow/flower_plot.

Python scripts were used to convert the table generated by Proteinortho into a binary presence-absence matrix of genes and to create an accumulation curve of the total pangenome and core genes (https://github.com/isabelschober/proteinortho_downstream). Heaps’ law was applied to the presence-absence matrix of genes to calculate the α value to predict whether the pangenome is open or closed. The calculation was performed in R using the Micropan package (9) in “random” mode with 1,000 permutations.

### Core gene phylogeny

Amino acid sequences for each of the identified core genes were aligned using MAFFT v7 (10) with the L-INS-i strategy. To enhance alignment quality, the resulting alignments were refined with Gblocks (11) to remove poorly aligned regions. These individual alignments were preserved for generating phylogenies of single core genes.

The alignments were then analyzed with IQ-TREE (12) to construct maximum likelihood phylogenetic trees. The LG+F+R4 substitution model, as recommended by ModelFinder (13) based on the Bayesian information criterion, was applied during tree construction. Branch support values (bootstrap) were calculated with 1,000 replicates. This step utilized resources from the Centro Nacional de Processamento de Alto Desempenho em São Paulo (CENAPAD-SP) and the Gloriosos clusters at the Laboratory of Genetics Biochemistry, UFMG.

To infer a consensus species tree while accounting for potential discordance among individual gene trees, we employed Weighted ASTRAL (wASTRAL v1.22.3.7) (14). This method improves phylogenomic inference by integrating both branch support values (bootstrap) and branch lengths to weight the contribution of quartets, effectively reducing the influence of low-confidence signals. Gene trees generated for each core gene by IQ-TREE were used as input. wASTRAL was executed with support-weighted and length-weighted hybrid criteria.

The phylogenetic tree was visualized in FigTree v1.4.4 (https://tree.bio.ed.ac.uk/software/figtree) and edited in Inkscape v1.2.1 (2022).

### Functional analysis of clustered predicted proteins

To predict the functions of clustered proteins, the amino acid sequences in their respective pangenomic groups were mapped to the Giant Virus Orthologous Groups (GVOGs) (15), a curated set of protein families specific to the *Nucleocytoviricota*. This mapping was performed using Hidden Markov Models (HMMs) with the *hmmscan* tool from the HMMER package (16). The *hmmscan* tool identifies the presence of conserved domains within protein sequences by comparing them to pre-built HMM profiles of known protein families. Subsequently, functional annotations were inferred from the GVOG descriptions, based on the Cluster of Orthologous Groups classification.

### Synteny analysis

The conservation of gene order is an important indicator of shared evolutionary history. For this analysis, the predicted protein sequences were used in an all-against-all BLASTp (1e-5). The resulting BLASTp table was filtered, selecting hits with p-ident ≥20% and ≥70%. MCScanX, a tool that detects gene collinearity in genome pairs (17), was then used for this purpose. MCScanX utilizes BLASTp outputs along with the GFF files generated by GeneMarkS containing the location of the predicted proteins to assess the presence of syntenic gene blocks among genome pairs. Two analyses were conducted, one for p-ident ≥20% and another for p-ident ≥70%. The results were used to create a synteny matrix, visualized and downloaded through Synvisio (18). The two matrices were then combined and edited in Inkscape v1.2.1.

### AAI and ANI calculation

To estimate the levels of similarity between genomes in terms of nucleotide and amino acid composition, the genomes and amino acid sequences, respectively, were submitted to the ANI/AAI calculator (19) to calculate average nucleotide identity (ANI) and average amino acid identity (AAI) values. This tool estimates the average identity of nucleotides and amino acids through BLAST using a bidirectional approach where best hits (one-way ANI/AAI) and reciprocal best hits (two-way AAI/ANI) between two sets of genomic protein data are computed.

Matrices were created with the pairwise values and submitted to R for heatmap generation with pheatmap v1.0.12 (20). Hierarchical clusters were then calculated with the hclust function in R, using the complete linkage method, and the genomes were reorganized into dendrograms with the dendsort package (21) and then plotted with ggplot2. For comparison purposes, average AAI values were also calculated for genomes from other NCLDV families (Table S1).

## GENOMIC DIVERSITY AND CONTENT PATTERNS REVEAL DISTINCT GROUPINGS WITHIN EXTENDED *ASFARVIRIDAE*

A search of the NCBI database for complete viral species genomes of extended *Asfarviridae* returned 39 complete genomes as of June 2023 (Table S2). All sequences were obtained in FASTA format. The analyzed attributes included genome length, architecture, and G + C content. Subsequently, the genomes were annotated for coding sequences using the GeneMarkS tool.

No statistically significant differences (Mann-Whitney *P* < 0.05) were found between the genome sizes of pacmanviruses and kaumoebaviruses, but significant divergences were found when comparing the other groups (Fig. S1A). It is noteworthy that there is a significant difference in genome length between ASFV and the faustoviruses, which possess the largest genomes within the clade. Faustoviruses have, on average, a genome 2.5 times larger than ASFV, which has the smallest genomic size in the group.

ASFV exhibits the lowest number of predicted proteins (Fig. S1B), whereas no statistically significant differences were observed among kaumoebaviruses, pacmanviruses, and faustoviruses. Although the genomes of kaumoebaviruses and pacmanviruses are slightly shorter, they still maintain a number of proteins similar to those of faustoviruses, the group with the largest average genome size.

Faustoviruses and ASFV showed no significant differences in G + C content among their members (Fig. S1C). AbALV has the lowest G + C content in the group, almost 40% lower than the highest G + C content found in kaumoebavirus Sc. Faustoviruses are divided into clear three ranges of G + C content: faustovirus D5a, E12, E23, E24, Liban, and ST1, with an average of 36.3%; faustovirus D3, D5b, D6, and VV10, with an average of 37.7%; and faustovirus E9, LCD7, M6, S17, vv57, VV63, with an average of 39.5%.

## EXTENDED *ASFARVIRIDAE* PRESENTS AN EXTENSIVE OPEN PANGENOME AND A LIMITED CORE

**Fig 1:**
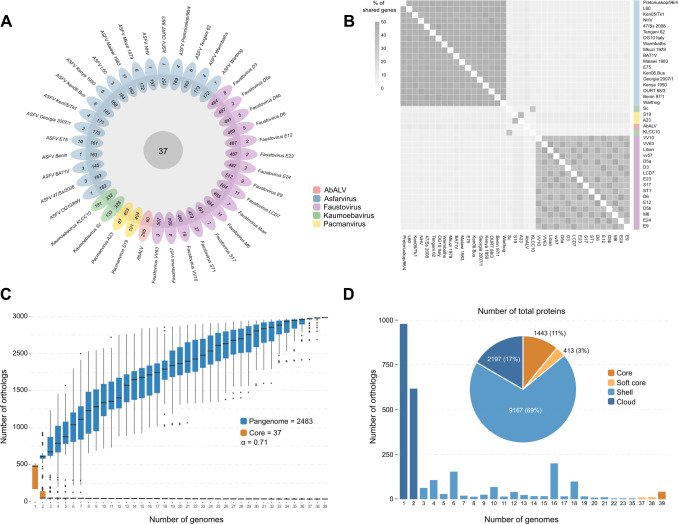
Pangenome structure and gene-sharing patterns in extended *Asfarviridae*. (**A**) Flower plot illustrating the distribution of core and singleton genes across the 39 genomes. Only 37 proteins are conserved across all genomes, while 973 are singletons, representing lineage-specific or unique genes. (**B**) Heatmap depicting the percentage of shared proteins between pairs of genomes, highlighting distinct gene-sharing patterns. (**C**) Gene accumulation curve for the total pangenome and core genes of extended *Asfarviridae*. The α value represents Heaps’ law power parameter for the average number of new genes per genome after 1,000 pangenome permutations. Values below 1 indicate an “open” pangenome state. (**D**) Histogram of gene distributions across the pangenomic groups: core, soft core, shell, and cloud. The pie chart displays the total number of genes in each category, with percentages in parentheses. AbALV: Abalone asfar-like virus; ASFV: African swine fever virus.

The clustering process of protein sequences based on similarity resulted in a total of 2,483 clusters of orthologous proteins or singleton genes for extended *Asfarviridae*. Only 37 proteins are found in all the 39 genomes, while 973 proteins were considered singletons (Fig. 1A), part of the cloud group, meaning they were not found in two or more genomes.

For each lineage analyzed, the core genes corresponded to approximately 7%–8% of the total genes in faustoviruses, kaumoebaviruses, and pacmanviruses. However, the scenario is different for AbALV, where core genes represent 10% of the total genes, and in ASFV, where this proportion varies from 20% to 25% of the genome.

Conversely, when examining singleton genes, we observe significant variation in proportions across different lineages. Faustoviruses and ASFV display relatively low proportions, ranging from 0 to 5% and 0 to 6%, respectively. In contrast, AbALV exhibits a notably high proportion of 83% singleton genes. Pacmanviruses and kaumoebaviruses occupy an intermediate range, with proportions between 14% and 20% and 29% and 35%, respectively.

Gene-sharing patterns within this clade exhibit distinct group-specific dynamics (Fig. 1B). Intragroup variation in ASFVs and faustoviruses ranges from 41% to 49% and 32% to 50%, respectively. Kaumoebaviruses, despite their genetic proximity, share only 33% of proteins, representing the highest intergroup sharing. Pacmanviruses share slightly more proteins (41%), while AbALV shows greater proximity to ASFVs (10%–11%). All other intergroup comparisons reveal less than 10% shared proteins.

The sequential inclusion of genomes does not clearly show the formation of a plateau at the end of the curve, suggesting that new inclusions can still increase the number of new genes (Fig. 1C). After applying power regression (n = κN−α) to the total number of genes in different lineage combinations, the parameter α was estimated as 0.71. Since α < 1 and no plateau is observed, the *Asfarviridae* pangenome is considered open. It is important to note that the core gene curve stabilizes quickly. Unlike the pangenome curve, this core gene group appears conserved with little variation as new genomes are included.

When stratifying orthologous protein clusters according to their pangenomic group, that is, according to the level of protein sharing, the observed patterns are illustrated in Fig. 1D. The histogram depicts the gene frequency of orthologs in each genome, while the pie chart illustrates the proportion of total redundant proteins in each pangenomic group. The histogram indicates that most genes are present in a limited number of genomes (accessory genes), while few genes are present in all genomes (core genes), suggesting a high variation in the gene content of viruses in this clade. Additionally, there is a predominance of singleton/cloud proteins in the clusters, although shell proteins lead in terms of the total number of proteins in the pie chart.

Protein lengths within this clade vary significantly by pangenomic group (Fig. S2). Core group proteins are the largest, surpassing both other virus groups and the average protein length across the entire pangenome. This group is followed by soft core, shell, and cloud proteins. Statistical analysis (Mann-Whitney, *P* < 0.05) confirms significant differences in mean lengths between these groups. Notably, cloud group proteins are consistently the smallest.

## CHALLENGES IN FUNCTIONAL ANNOTATION AND MCP FRAGMENTATION IN EXTENDED *ASFARVIRIDAE*

Functional annotation was achieved for 100% of the orthologous clusters in the core and soft-core groups, while the shell and cloud groups showed lower annotation rates of 60% and 55%, respectively, likely reflecting the abundance of singleton genes in the latter. Proteins of unknown function represent the most common category across all groups, according to the GVOG classification (Fig. S3).

As expected, most of the identified core proteins are functionally associated with DNA maintenance processes, including replication, reinforcing the essential role of these functions in the core genome of the extended *Asfarviridae*. In the soft-core group, the most frequent category is associated with transcription, replication, recombination, and repair, emphasizing the continued importance of these processes even in a less conserved set of genes. The shell group exhibited the greatest functional diversity, with more functions related to metabolism and cellular processes beyond nucleic acids. Aside from the significant proportion of unknown functions, the most represented categories in the shell group are energy production and conversion, secondary metabolite biosynthesis, transport and catabolism, and cell wall/membrane/envelope biogenesis. In the cloud group, the predominant categories are replication, recombination, and repair, and secondary metabolite biosynthesis, transport, and catabolism.

The absence of the structural protein Major Capsid Protein (MCP) in the results is noteworthy. MCP plays a crucial role in forming the viral capsid and is typically present in all viral genomes within the *Bamfordvirae* kingdom, which includes the NCLDVs. However, in faustoviruses, this gene is known to be fragmented across the genome (22), potentially impacting the clustering step. To confirm MCP’s presence in faustoviruses, a targeted search using BLASTp was conducted, locating 43 transcripts corresponding to MCP in faustoviruses, with an average of 2.6 transcripts per genome. These genes were then aligned with MCP sequences from other members of the group using MAFFT v7 (L-INS-i strategy), and the resulting multiple alignment was visualized in AliView (23) (Fig. S4).

## PHYLOGENETIC ANALYSIS REVEALS HIGH GENETIC DIVERGENCE AMONG EXTENDED *ASFARVIRIDAE* GROUPS

**Fig 2:**
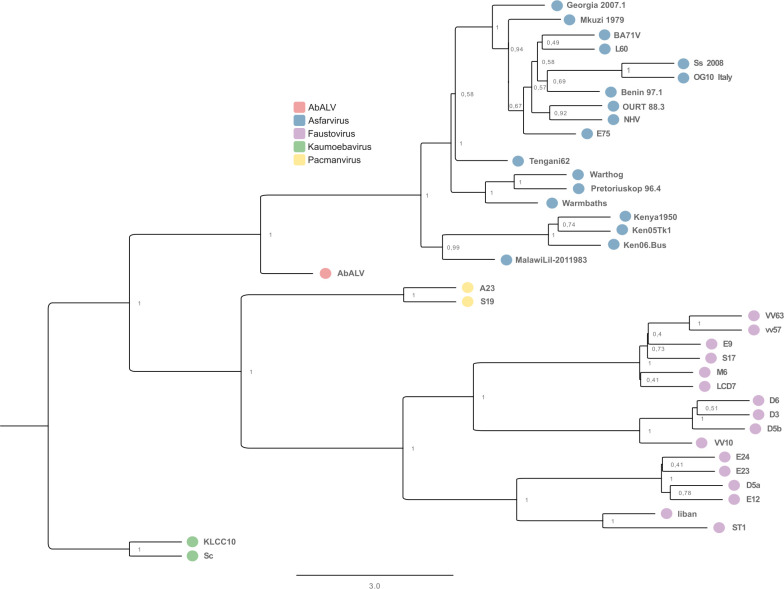
Phylogenetic relationships within the extended *Asfarviridae* based on core genes. Species tree reconstructed using the weighted ASTRAL (wASTRAL) method from individual maximum likelihood (ML) core gene trees, incorporating hybrid support based on bootstrap values and branch lengths. AbALV: Abalone asfar-like virus; ASFV: African swine fever virus.

The species tree (Fig. 2) was inferred using wASTRAL, based on 37 maximum likelihood core gene trees of the extended *Asfarviridae* group. This coalescent-based approach accounted for potential topological discordance among individual gene trees by integrating both branch support and branch length weighting. The resulting tree, rooted using kaumoebavirus as the basal lineage, revealed a well-resolved phylogenetic structure, with strong support for major clades. Faustovirus, pacmanvirus, ASFV, AbALV, and kaumoebavirus each formed strongly supported monophyletic groups, with high local posterior probabilities across internal nodes. Despite the shared ancestry among these viruses, the patristic distances separating the major clades were pronounced, typically exceeding 2.0 and reaching over 3.0 in several cases, indicating deep evolutionary divergence within the group.

## SYNTENY ANALYSIS REVEALS VARIATIONS IN GENE ORDER CONSERVATION

**Fig 3:**
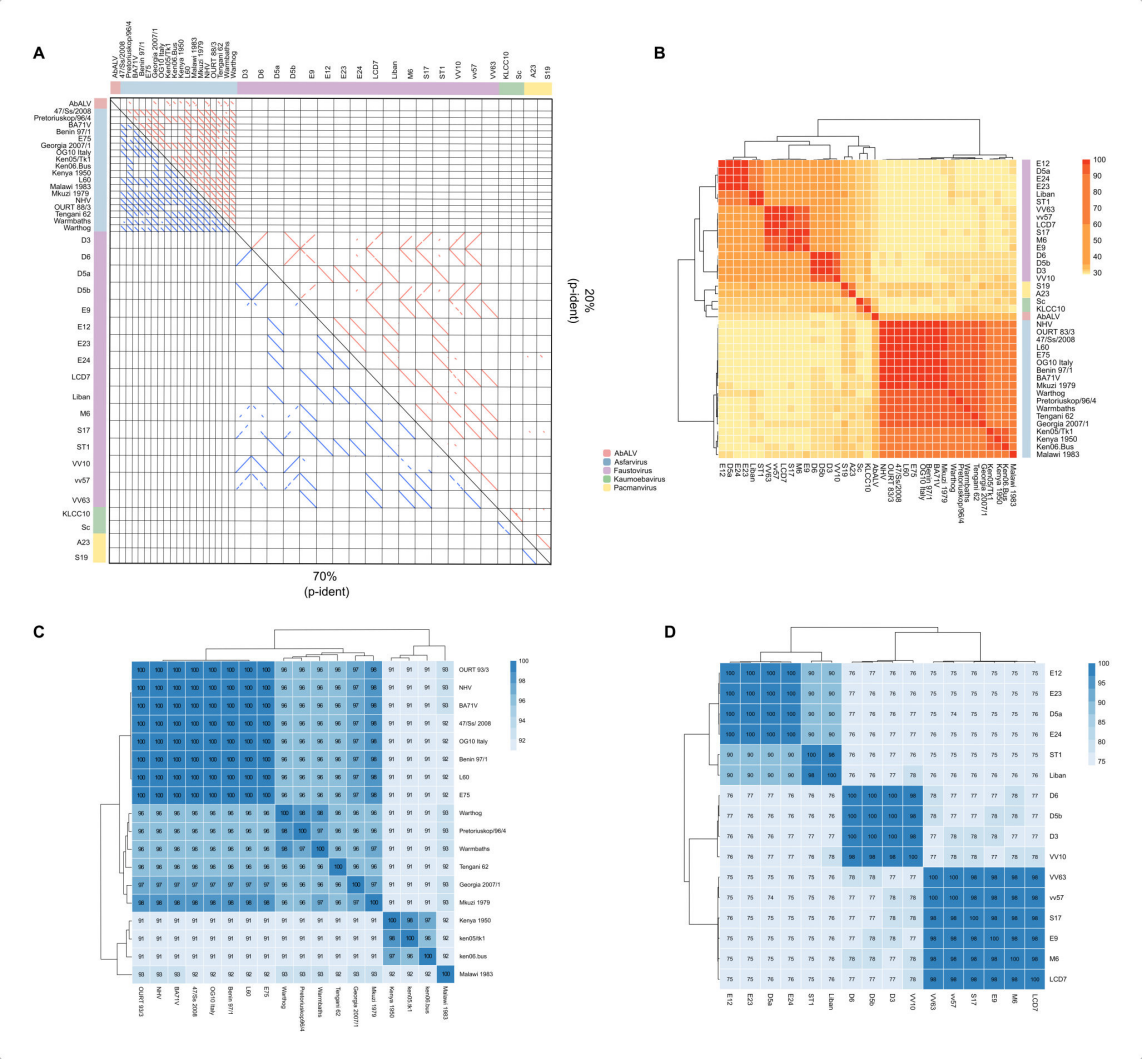
Comparative synteny and sequence identity reveal taxonomic boundaries in extended *Asfarviridae*. (**A**) Synteny analysis of 39 members of the extended *Asfarviridae*. Blue lines indicate a minimum identity of 70%, while red lines indicate a minimum identity of 20%. Each block represents a pairwise collinearity comparison between two viruses. White blocks indicate the absence of collinear amino acid sequences. (**B**) Heatmap of pairwise AAI values among 39 genomes. The color scale is segmented by quartiles of AAI values. Hierarchical clustering was performed using the Ward method with Euclidean distance. (**C**) Heatmap of pairwise ANI values for 18 ASFV genomes. (**D**) Heatmap of pairwise ANI values for 16 faustovirus genomes. Hierarchical clustering was performed using the Ward method with Euclidean distance. Organisms with ANI above 95% are frequently considered the same species.

Synteny analyses using dot plots provide a comprehensive view of genomic organization. BLASTp was used at two identity thresholds (20% and 70%) alongside MCScanX, a tool for detecting synteny. The results showed that gene order conservation varies for each virus group, even with reduced protein similarity. Intergroup comparisons revealed that only AbALV and ASFV showed gene collinearity through segmented gene blocks in the genome (Fig. 3A), especially with reduced protein similarity (p-ident).

Within groups, ASFVs demonstrated high gene order conservation, except for the group formed by ASFV BA71V, Benin 97/1, and E75, which showed no collinearity with the ASFV ken05/tk1, Kenya 1950, and ken06.Bus group. In contrast, faustoviruses showed variations in gene order conservation, ranging from a complete absence to full conservation, depending on the group members. In faustoviruses, genome sequence inversion is observed, as seen in diagonal lines from the bottom left to the top right. White boxes indicate a lack of similarity between the analyzed pairs.

## ANI AND AAI VARIABILITY DELINEATES POTENTIAL NEW SPECIES AND GENERA WITHIN EXTENDED *ASFARVIRIDAE*

Analyses of AAI and ANI were performed to compare the amino acid and nucleotide compositions between different viral specimens. The AAI matrix (Fig. 3B) highlights proximity and divergence between groups. Hierarchical clustering revealed distinct patterns of similarity, with ASFVs showing high internal AAI values (>95%) and significantly lower values (~30%) when compared to other groups. AbALV is the closest to ASFVs, with an AAI of 32%. Among faustoviruses, four clusters with high AAI (>95%) were identified: (i) E12, D5a, E23, and E24; (ii) ST1 and Liban; (iii) E9, M6, S17, LCD7, vv57, and VV63; and (iv) D6, D5b, D3, and VV10. AAI values between faustovirus clusters drop to 50%–60%, and comparisons with other groups are around 30%.

Pacmanviruses and kaumoebaviruses exhibited high AAI similarity within their respective groups, with kaumoebaviruses at 63% AAI and pacmanviruses at 83%. Comparisons between these viruses and ASFV, faustoviruses, and AbALV yielded lower AAI values, ranging from 29% to 35%. These AAI results suggest a closer relationship among faustoviruses, kaumoebaviruses, and pacmanviruses, as compared to ASFV and AbALV, while highlighting notable intragroup variation. The average AAI among the 39 members of the extended *Asfarviridae* was 37%, exceeding that of *Ascoviridae* (34% ± 3.49%), *Iridoviridae* (34% ± 4.37%), and *Marseilleviridae* (37% ± 8.07%), but lower than *Mimiviridae* (44% ± 13.22%). However, the extended *Asfarviridae* exhibit greater intra-group variability.

ANI analyses were performed individually due to methodological limitations with highly divergent groups. Organisms with over 95% ANI are frequently considered to belong to the same species. ASFV subgroups identified include the following: (i) ASFV Malawi 1983; (ii) ASFV ken05/tk1, Kenya 1950, ken06.Bus; (iii) ASFV Georgia 2007/1, Mkuzi 1979, Tengani 62, Warmbaths, Pretoriouskop/96/4, and Warthog; and (iv) other ASFVs, all with ANI above 95% (Fig. 3C).

Faustoviruses demonstrate higher divergence in ANI compared to ASFVs, with four distinct groups identified in both AAI and ANI analyses, each showing intragroup ANI above 98% (Fig. 3D), indicating the likely presence of four separate species within this group. Intergroup comparisons between faustoviruses and other viral groups reveal ANI values below 78%. Kaumoebavirus strains KLCC10 and Sc show a low ANI of 77.93%, while pacmanvirus strains A23 and S19 exhibit a slightly higher ANI at 85.64%, suggesting closer genomic similarity among pacmanviruses than kaumoebaviruses, although both comparisons fall below the 95% ANI threshold. These findings further support the presence of two distinct species within both the pacmanvirus and kaumoebavirus groups.

## DISCUSSION

The term “extended *Asfarviridae”* emerged as a temporary classification to encompass a variety of giant viruses, including AbALV, faustovirus, kaumoebavirus, and pacmanvirus, as well as potential other viruses yet to be discovered that exhibit some level of phylogenetic similarity with ASFV, the original member of this clade. The inclusion of these new members results in a broad biological diversity within the extended *Asfarviridae*. These viruses infect a wide range of hosts, from unicellular amoebae to multicellular organisms such as mollusks and vertebrates. While Asfarvirus (ASFV) and AbALV occur naturally in their respective hosts, the true host range of amoeba-associated members (e.g., *faustovirus*, *kaumoebavirus*, *pacmanvirus*) remains uncertain, as the reported hosts may represent systems of isolation.

Our genomic analysis highlights variability in features such as genome length, G + C content, and the number of predicted proteins, which aid in viral classification. Although G + C content is traditionally used in taxonomic descriptions, often varying by less than 1% between species (24), our findings, consistent with Witt et al. (25), suggest that it is not a reliable taxonomic marker at the family level within *Nucleocytoviricota*. Only *Marseilleviridae* shows notable uniformity in this aspect. Within the extended *Asfarviridae*, faustoviruses are the closest group to ASFV, while others show greater divergence. Additionally, the division of faustovirus genomes into three distinct G + C content bands supports the presence of subgroups (3). Protein-coding patterns reflect that faustoviruses, pacmanviruses, and kaumoebaviruses each display a relatively stable gene count (26), whereas ASFV carries far fewer genes, reflecting its smaller genome. Crucially, comparable protein numbers can accompany different genome lengths, highlighting variable gene density. Yet, within an established viral family, genome size itself tends to remain consistent (27, 28). In our analyses, viruses infecting unicellular eukaryotes (e.g., *Vermamoeba, Acanthamoeba*) cluster together and are genetically closer to each other, whereas those infecting multicellular hosts (e.g., abalones, swine) form a distinct, more compact group with smaller genomes. This pattern may reflect genome streamlining or gene loss associated with adaptation to more specialized host environments.

A previous analysis of the extended *Asfarviridae* pangenome by Karki et al. (29) primarily focused on investigating aquatic metagenomic-assembled genomes, without stratifying the pangenome into its various groups or calculating whether the pangenome was open or closed. We addressed this gap by applying the Heaps formula, proposed by Tettelin et al. (30, 31), and by constructing a pangenomic accumulation curve to evaluate this aspect. The results indicate that the extended *Asfarviridae* pangenome is considered open. This categorization implies that the discovery and incorporation of new genomes tend to add new genes to the pangenome, suggesting that the diversity of extended *Asfarviridae* is not yet fully known. Unlike many viral groups whose pangenomes are closed due to the limited number of genes, NCLDVs may have genomes without this limitation, resulting in open pangenomes, as observed in this study. Examples of other viral groups with open pangenomes include pandoraviruses (32), the *Herpesviridae* family (33), and ASFVs at the species level (34).

The core genome of the extended *Asfarviridae* stabilizes rapidly at only 37 genes, a size close to the 47-gene ancestral NCLDV core (35) yet far below the 86-gene core of the more cohesive *Asfivirus* genus (34). This minimal, highly conserved set, set against a pangenome of 2,483 orthologous clusters, underscores the deep genomic divergence within the clade and suggests the core would expand if its members were more closely related. By comparison, other giant viruses retain much larger cores, such as the pandoravirus group ≈425 genes (15%–30% of each genome) and the families *Mimiviridae* 267 genes (22%–28%) and *Marseilleviridae* 202 genes (~44%) (32, 36, 37). Even the debated pithovirus clade (cedratvirus + orpheovirus) carries 52 core genes (38). Thus, extended *Asfarviridae* appears to sit near the minimal core boundary of NCLDVs, highlighting its exceptional heterogeneity. Core group proteins also tend to be longer than accessory ones, a feature linked to functional and evolutionary importance and possibly reflecting a more ancient origin (39). Natural selection is known to suppress changes in longer transcripts while promoting variation in shorter ones (40), which reinforces the stability of this minimal set. In contrast, cloud proteins are generally smaller, more prone to accumulate mutations, and likely represent younger, accessory functions, a pattern also observed in eukaryotes (41) and now evident in viruses.

A significant portion of proteins in the extended *Asfarviridae* pangenome lacks known functions, a recurring issue in NCLDV genomes, where over 80% of proteins can be uncharacterized (42). The core group has the highest proportion of identified proteins due to its conservation and essential genes, aiding functional identification. Core group genes, vital for basic biological functions, include many NCLDV housekeeping genes (35). The MCP gene, critical for all extended *Asfarviridae*, is absent from the core group due to fragmentation in faustoviruses and divergence in other viruses (22). This absence is not uncommon in core genomes; in various studies, it is also not detected in pandoraviruses and some divergent pithoviruses (42). This reflects a limitation of pangenomic techniques in detecting atypical gene structures. Other pangenomic groups showed limited functional annotation due to the high proportion of unknown proteins.

Although these viruses share a common ancestry, the species tree inferred from multi-gene coalescent analysis revealed that kaumoebavirus, faustovirus, pacmanvirus, ASFV, and AbALV each form a highly supported and genetically cohesive clade. The patristic distances, that is, the summed branch lengths between taxa, separating these lineages were consistently high, reflecting levels of divergence comparable to those observed among the major clades in this data set. This degree of evolutionary separation, combined with robust monophyly and long internal branches, is inconsistent with a single-family classification.

To further investigate the taxonomic implications of these phylogenetic patterns, we integrated multiple genomic analyses, including phylogeny, AAI, ANI, and synteny. These analyses clearly support the delineation of multiple species within the extended *Asfarviridae*. The clade-wide mean AAI, a key metric for taxonomic classification, is just 37%, yet ASFV isolates share >95% AAI and minor syntenic differences, supporting three species: (i) ken05/tk1, Kenya 1950, and ken06.Bus; (ii) Malawi 1983; and (iii) the remaining 14 isolates. Faustoviruses resolve into four species based on congruent AAI/ANI values and phylogenetic topology: (i) E12, D5a, E23, E24; (ii) ST1 and Liban; (iii) E9, M6, S17, LCD7, vv57, VV63; and (iv) D6, D5b, D3, VV10. Pacmanviruses and kaumoebaviruses likely each encompass two species. AbALV lies below the *Asfivirus* AAI threshold, occupying an intermediate position. Collectively, these findings reveal substantial genomic divergence within the extended *Asfarviridae*, indicating evolutionary separations that support taxonomic revision.

Altogether, these findings suggest that these aggregated viruses likely do not belong to a single taxonomic family, especially when compared to the only official member, the *Asfivirus* genus. Instead, faustoviruses, kaumoebaviruses, pacmanviruses, and Abalone asfar-like virus represent a cluster of related yet genetically diverse species that may warrant classification into distinct families. Based on our analysis, the 16 faustoviruses likely comprise four species, the 18 ASFV three species, and the pairs of pacmanviruses and kaumoebaviruses two species each. The open nature of the pangenome underscores the vast unexplored diversity of these viruses. Additional viral sequences and further studies are essential to deepen our understanding of this clade’s genomic complexity and its relationships with hosts.

### Taxonomic implications

Based on the strong phylogenetic, genomic, and pangenomic evidence presented, we propose the formal taxonomic reclassification of the so-called extended *Asfarviridae* clade. The deep divergence between ASFV, faustoviruses, kaumoebaviruses, pacmanviruses, and AbALV, evidenced by consistently low intergroup AAI values (29%–35%), high patristic distances, and limited gene sharing, exceeds the thresholds typically observed between established viral families. These lineages form well-supported, monophyletic clades with intra-group AAI and ANI values above 95% (consistent with species-level cohesion), while intergroup AAI falls below 40% (supporting family-level separation).

Following established thresholds for *Nucleocytoviricota* taxonomy and the ICTV’s genome-based criteria, we propose that the extended *Asfarviridae* be reclassified into five distinct families:

- *Asfarviridae* (retained; genus *Asfivirus*, 3 species),
- *Faustoviridae* fam. nov. (4 species across 4 subclades for all faustovirus species),
- *Kaumoebaviridae* fam. nov. (2 genera, 2 species for kaumoebavirus),
- *Pacmanviridae* fam. nov. (2 genera, 2 species for pacmanvirus),
- *Abaloneviridae* fam. nov. (genus *Abalonevirus*; pending additional isolates).

### Demarcation criteria

Our proposal adheres to the quantitative and evolutionary benchmarks presented in Table 1.

**TABLE 1:** Proposed demarcation criteria for taxonomic classification within the extended *Asfarviridae^a^*

Taxonomic rank	Genomic criteria	Evolutionary criteria
Species	ANI ≥ 95% across >75% of genes	Conserved synteny blocks
Genus	AAI 40–70% + functional divergence (e.g., annotation profiles, gene density shifts)	Monophyly (local PP > 0.80)
Family	AAI < 40% between groups + distinct core genome size	High patristic distance (>2.0) +concordant monophyly (bootstrap > 80%)

^*a*^ Quantitative genomic thresholds and evolutionary benchmarks used to define species, genus, and family ranks based on comparative analyses.

This proposal is based on multiple complementary criteria:

- Monophyly with high branch support (>0.95 local posterior probabilities),
- AAI values between groups below 40%,
- ANI values between genera consistently below 70%,
- Patristic distances consistent with or exceeding those between established NCLDV families such as *Marseilleviridae*, *Iridoviridae*, and *Mimiviridae*.

Furthermore, each proposed family exhibits lineage-specific genomic features, including genome length, G + C content, pangenome structure, and functional gene content, that support their distinction at higher taxonomic ranks. These findings reinforce the need to revise the taxonomy of this clade and align with the ICTV’s current standards for sequence-based virus classification.

## SUPPLEMENTAL MATERIAL

Supplemental material jvi.01337-25-s0001.pdf Tables S1 and S2; Fig. S1 to S4.

ASM does not own the copyrights to Supplemental Material that may be linked to, or accessed through, an article. The authors have granted ASM a non-exclusive, world-wide license to publish the Supplemental Material files. Please contact the corresponding author directly for reuse.
