# Supplementary material for: Integrative genomic analyses support the division of the extended Asfarviridae clade into multiple viral families
Source: J Virol. 2025 Nov 13;99(12):e01337-25. doi: 10.1128/jvi.01337-25 (PMC12724278; doi:10.1128/jvi.01337-25)
Supplement: Supplemental material — Tables S1 and S2; Fig. S1 to S4. [file jvi.01337-25-s0001.pdf]

Integrative Genomic Analyses Support the Division of the Extended *Asfarviridae* Clade into Multiple Viral Families.

Thiago Mendonça dos Santos, Jônatas Abrahão & Luiz-Eduardo Del-Bem.

Supplemental Material

Supplemental Table 1. Genomes from other NCLDV families used for AAI comparison.

Family	Genomes
Iridoviridae	*Lymphocystivirus platichthys, Megalocytivirus lates, Megalocytivirus pagrus, Ranavirus rana, Iridovirus armadillidium, Iridovirus chilo, Chloriridovirus wiseana*, *Decapodiridovirus litopenaeus*
Marseilleviridae	*Losannavirus lausannense, Losannavirus tunisense, Marseillevirus massiliense*, golden marseillevirus, brazilian marseillevirus, tokyovirus A1
Ascoviridae	Toursvirus dptv1a, Ascovirus sfav1a, Ascovirus hvav3a, Ascovirus tnav2a
Mimiviridae	*Mimivirus bradfordmassiliense*, *Theiavirus salishense, Rheavirus sinusmexicani, Cotonvirus japonicum, Fadolivirus algeromassiliense, Megavirus chiliensis, Moumouvirus australiensis, Moumouvirus goulettemasseliense, Megavirus powaiense, Tupanvirus altamarinense, Tupanvirus salinum*, Yellowstone lake mimivirus

Supplemental Table 2. Complete genomes of extended *Asfarviridae* used in this study.

Group	Strains (NCBI Accession Numbers)
ASFV (18)	ASFV OG10 Italy (NC_044947.1), ASFV 47/ss/2008 (NC_044955.1), ASFV BA71V (NC_001659.2), ASFV Benin 97/1 (NC_044956.1), ASFV E75 (NC_044958.1), ASFV Georgia 2007/1 (NC_044959.2), ASFV ken05/TK1 (NC_044945.1), ASFV ken06.Bus (NC_044946.1), ASFV Kenya 1950 (NC_044944.1), ASFV L60 (NC_044941.1), ASFV Malawi 1983 (NC_044954.1), ASFV Mkuzi 1979 (NC_044953.1), ASFV NHV (NC_044943.1), ASFV OURT 88/3 (NC_044957.1), ASFV Pretoriuskop/96/4 (AY261363.1), ASFV Tengani 62 (NC_044951.1), ASFV Warmbaths (NC_044950.1), ASFV Warthog (NC_044949.1)
Faustoviruses (16)	Faustovirus D3 (KU556803.1), Faustovirus D5a (KU702950.1), Faustovirus D5b (KU702949.1), Faustovirus D6 (KU702951.1), Faustovirus E12 (KJ614390.1), Faustovirus E23 (KU702952.1), Faustovirus E24 (KU702948.1), Faustovirus E9 (MT335755.1), Faustovirus LCD7 (MN830294.1), Faustovirus liban (MN534311.1), Faustovirus M6 (MN830295.1), Faustovirus S17 (MN830296.1), Faustovirus ST1 (LT839607.1), Faustovirus VV10 (MN956669.1), Faustovirus vv57 (MN830297.1), Faustovirus VV63 (MN830298.1)
Kaumoebaviruses (2)	Kaumoebavirus KLCC10 (MT334784.1), Kaumoebavirus Sc (NC_034249.1)
Pacmanviruses (2)	Pacmanvirus A23 (NC_034383.1), Pacmanvirus S19 (MZ440852.1)

AbALV (1)	AbALV (LC637659.1)

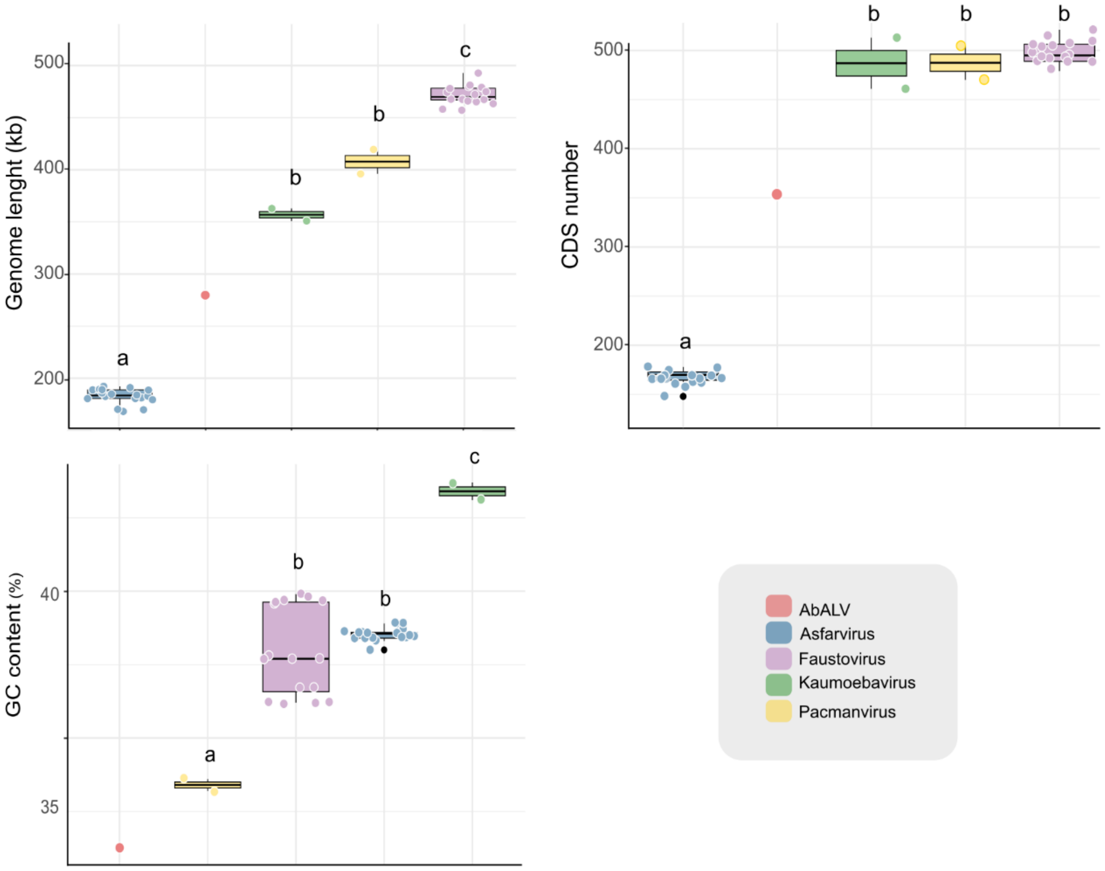

Supplemental Figure 1 – Comparative genomic features among members of the extended *Asfarviridae* family. (A) Genome size; (B) Number of predicted proteins; (C) G+C content. AbALV – Abalone asfar-like virus; ASFV – African Swine Fever Virus.

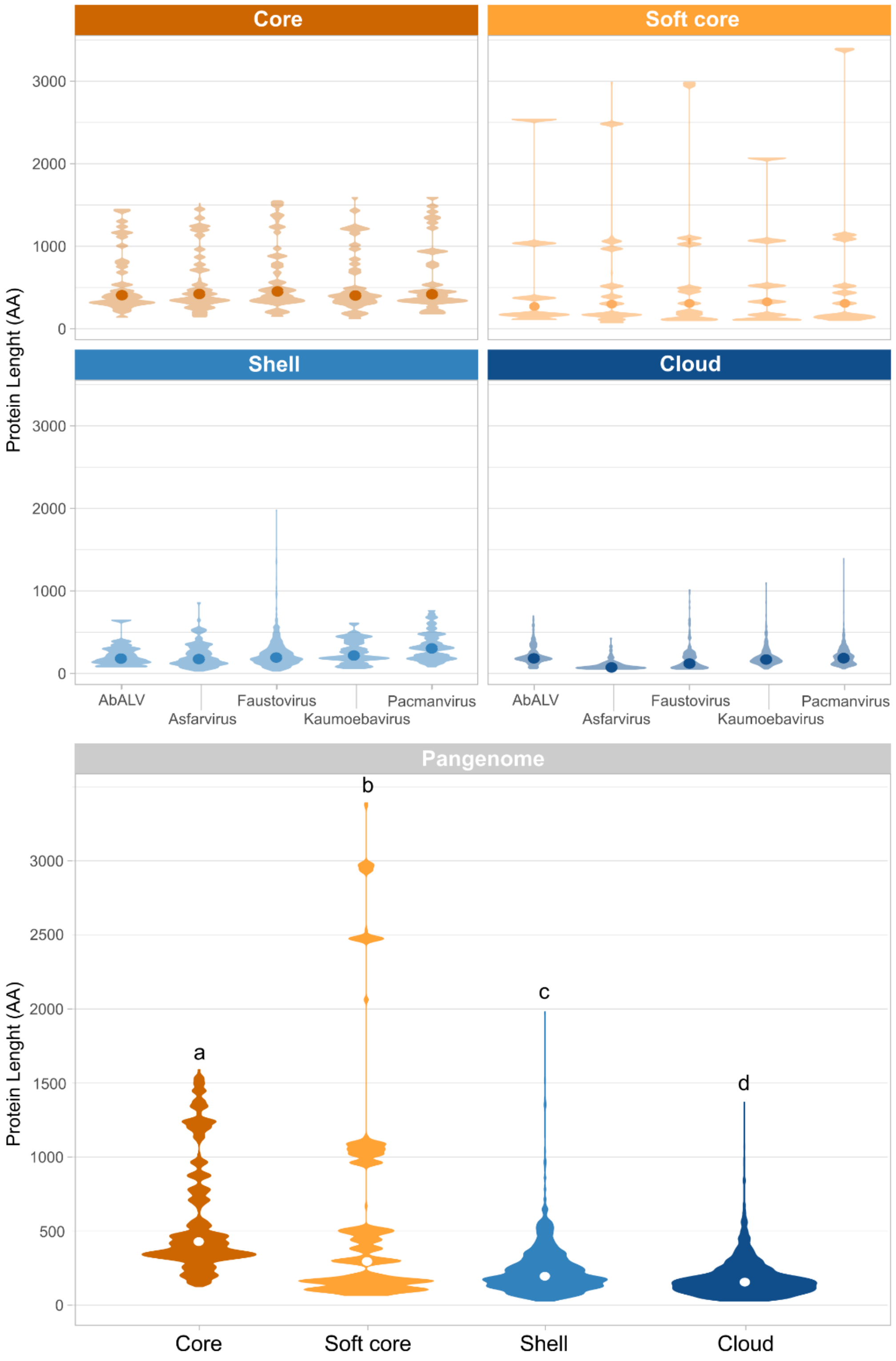

Supplemental Figure 2 – Protein length variation in the extended *Asfarviridae* pangenome, separated by pangenomic group and total. Different lowercase letters indicate statistically significant differences (Mann–Whitney, $p < 0.05$). AbALV – Abalone asfar-like virus.

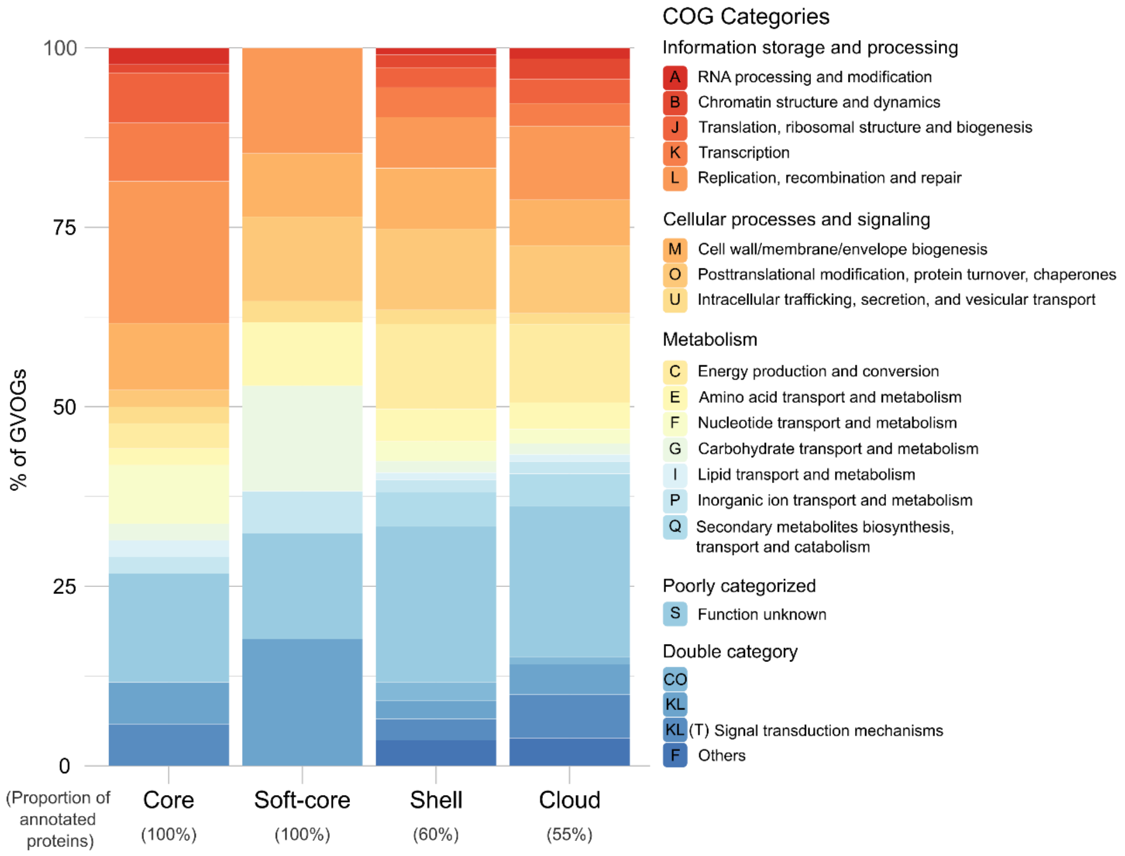

Supplemental Figure 3 – Functional classification of orthologous protein clusters in the extended *Asfarviridae* pangenome based on the GVOG database. All predicted proteins were aligned to the GVOG database to infer their functions. The uppercase letters on the Y-axis represent GVOG functional categories, as indicated in the legend. The percentage below each pangenome group indicates the proportion of annotated genes within the total number of genes in that group.

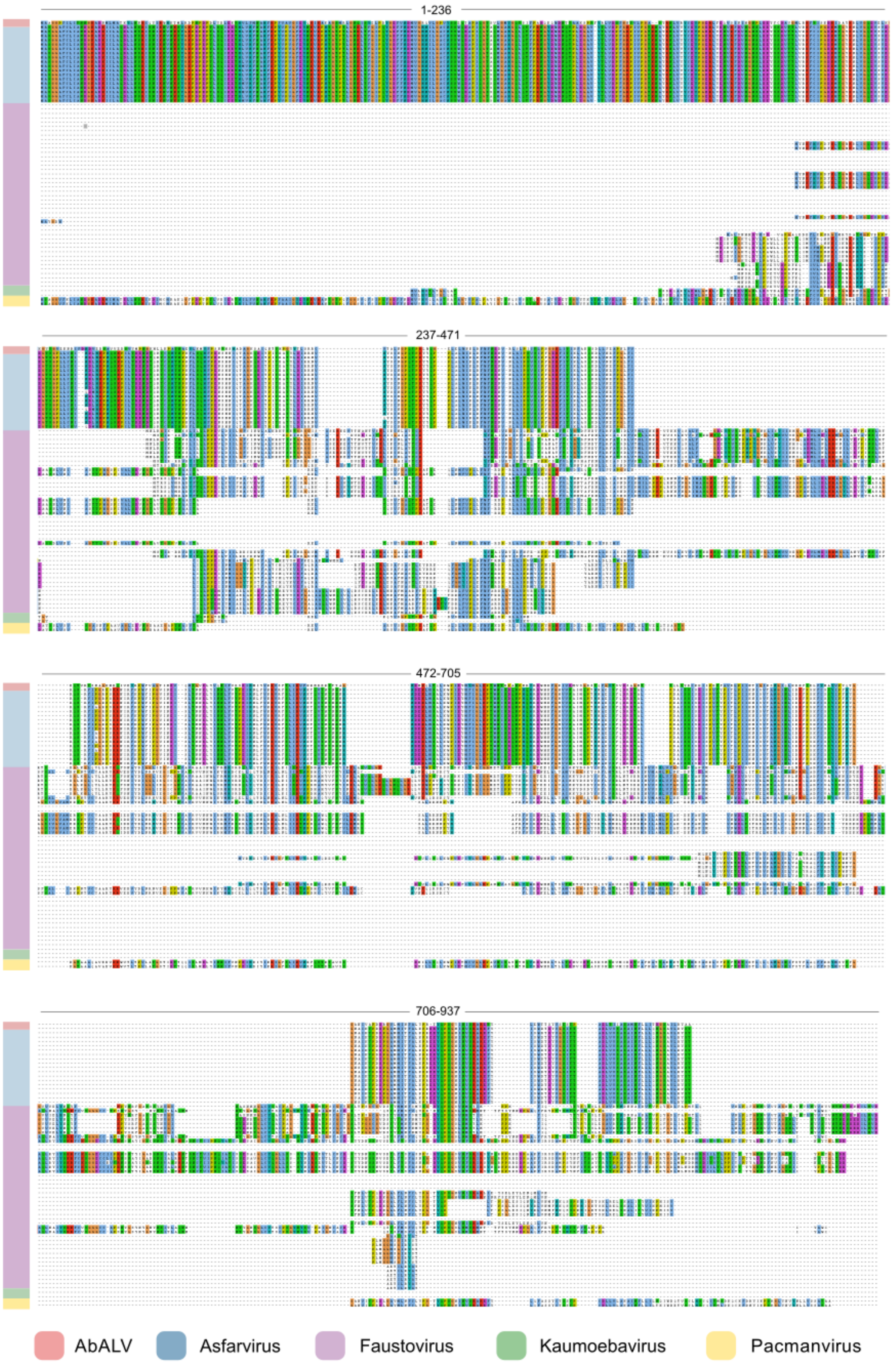

Supplemental Figure 4 - Multiple sequence alignment of Major Capsid Protein (MCP) sequences across the extended *Asfarviridae*. This alignment includes complete MCP sequences from ASFV, kaumoebavirus, pacmanviruses, AbALV, and fragmented MCP sequences from faustoviruses.

The faustovirus MCPs, previously undetected in orthologous clustering due to gene fragmentation, were identified through targeted BLASTp searches and aligned using MAFFT v7 (L-INS-i strategy). AbALV – Abalone asfar-like virus.
